# Alzheimer’s Disease and Related Dementias III A Systematic Review of Third Wave Cognitive Behavioral Interventions for People Diagnosed with Dementia

**DOI:** 10.1093/geroni/igaf122.2843

**Published:** 2025-12-31

**Authors:** Elizabeth Fauth, Ty Aller, Heather Kelley, Marissa Donahue

**Affiliations:** Utah State University, Logan, Utah, United States; Utah State University, Logan, Utah, United States; Utah State University, Logan, Utah, United States; Utah State University, Logan, Utah, United States

## Abstract

Millions of people receive a diagnosis of Alzheimer’s disease or related dementia (ADRD) each year, and increased depression and anxiety often co-occur. Third wave cognitive behavioral therapies (CBT) refer to a cluster of interventions based in CBT but which focus on improving psychological and behavioral processes as a whole, rather than focusing on symptom-reduction, although symptom-reduction is a common outcome. There is extensive research on the efficacy of third wave approaches in multiple populations, but there are limited studies of its efficacy and acceptability individuals with dementia. This study is systematic review of third-wave CBT for individuals with ADRD published between 2012 and 2023. Ten studies met inclusion criteria, of which 7 were unique studies and 3 were supplemental studies using a similar dataset. There were varying designs (e.g., single-arm, randomized control trial). A total of 229 participants with ADRD were included. Mindfulness-based interventions were most used (k = 3) and interventions were primarily delivered via group sessions. Adaptations for ADRD included shortening session intensity (e.g., fewer sessions, fewer activities) and making materials more accessible (e.g., larger fonts, fewer words). The most common outcome assessed was quality of life. Results of studies were mixed; some reported improvements while others reported null or negative outcomes. We conclude that third wave behavioral interventions seem to be preliminarily feasible and acceptable for individuals with dementia, but this research is still in the early stages. Individuals with ADRD need support in their mental health. Future research should emphasize more rigorous study design and increased sample sizes.

